# Human Hookworm Infection Enhances Mycobacterial Growth Inhibition and Associates With Reduced Risk of Tuberculosis Infection

**DOI:** 10.3389/fimmu.2018.02893

**Published:** 2018-12-14

**Authors:** Matthew K. O'Shea, Thomas E. Fletcher, Julius Muller, Rachel Tanner, Magali Matsumiya, J. Wendi Bailey, Jayne Jones, Steven G. Smith, Gavin Koh, William G. Horsnell, Nicholas J. Beeching, James Dunbar, Duncan Wilson, Adam F. Cunningham, Helen McShane

**Affiliations:** ^1^Nuffield Department of Medicine, The Jenner Institute, University of Oxford, Oxford, United Kingdom; ^2^Institute of Microbiology and Infection, University of Birmingham, Birmingham, United Kingdom; ^3^Royal Centre for Defence Medicine, Joint Medical Command, Birmingham, United Kingdom; ^4^Department of Clinical Sciences, Liverpool School of Tropical Medicine, Liverpool, United Kingdom; ^5^Department of Immunology and Infection, London School of Hygiene and Tropical Medicine, London, United Kingdom; ^6^Department of Infectious Diseases, Northwick Park Hospital, London, United Kingdom; ^7^Institute of Infectious Disease and Molecular Medicine and Division of Immunology, University of Cape Town, Cape Town, South Africa; ^8^Department of Infectious Diseases, The Friarage Hospital, Northallerton, United Kingdom; ^9^Institute of Immunology and Immunotherapy, University of Birmingham, Birmingham, United Kingdom

**Keywords:** tuberculosis, latent tuberculosis, LTBI, hookworm, eosinophil, growth inhibition

## Abstract

Soil-transmitted helminths and *Mycobacterium tuberculosis* frequently coincide geographically and it is hypothesized that gastrointestinal helminth infection may exacerbate tuberculosis (TB) disease by suppression of Th1 and Th17 responses. However, few studies have focused on latent TB infection (LTBI), which predominates globally. We performed a large observational study of healthy adults migrating from Nepal to the UK (*n* = 645). Individuals were screened for LTBI and gastrointestinal parasite infections. A significant negative association between hookworm and LTBI-positivity was seen (*OR* = 0.221; *p* = 0.039). Hookworm infection treatment did not affect LTBI conversions. Blood from individuals with hookworm had a significantly greater ability to control virulent mycobacterial growth *in vitro* than from those without, which was lost following hookworm treatment. There was a significant negative relationship between mycobacterial growth and eosinophil counts. Eosinophil-associated differential gene expression characterized the whole blood transcriptome of hookworm infection and correlated with improved mycobacterial control. These data provide a potential alternative explanation for the reduced prevalence of LTBI among individuals with hookworm infection, and possibly an anti-mycobacterial role for helminth-induced eosinophils.

## Introduction

Tuberculosis (TB) continues to be a major global health challenge despite reductions in prevalence and mortality in the last 25 years. In addition to the significant morbidity and mortality attributed to TB disease, recent estimates suggest that one quarter of the world's population is latently infected with *Mycobacterium tuberculosis* (*M.tb*) (1, 2). On a similar scale, it has been estimated that up to one third of the global population is infected with intestinal helminths (3–6). Soil-transmitted helminths and *M.tb* frequently coincide geographically and between 20 and 35% of people with TB disease are thought to be co-infected with helminths in high burden regions, particularly in resource-poor settings (7).

Despite the significant spatial overlap and the apparent prevalence of TB disease and helminth co-infection, there is limited understanding of the relationship between these pathogens. Helminths are commonly thought to modify the host immune response through modulation of the Th1-Th2 immune axis and the induction of molecules such as IL-10 and TGF-β, associated with a regulatory phenotype. Control of *M.tb* infection requires pro-inflammatory Th1 and Th17 responses, whilst helminth infection induces strong Th2 anti-inflammatory responses, which may suppress Th1 profiles (8–10). As a result it has been suggested that in individuals with *M.tb* infection, helminth-induced immunomodulation promotes progression to active TB disease, exacerbates TB pathology, and attenuates the efficacy of anti-TB therapy and BCG vaccination (11–14).

However, data from human and animal studies have failed to support the hypothesis that helminth infection has a negative impact on either TB disease or cellular immune responses to a candidate TB vaccine (15–22). In addition, despite the significant burden of co-infection, there are few studies investigating the association between helminths and latent TB infection (LTBI), or the impact of helminths on the acquisition of *M.tb*.

Furthermore, a small body of work suggests that helminth infections may influence interpretation of interferon-gamma (IFN-γ) release assays (IGRAs) used to diagnose LTBI, which rely on Th1-mediated immune responses (23–26). Such an influence may be skewing our understanding of the prevalence of *M.tb* infection and warrants further investigation.

Our objective was to perform a longitudinal cohort study investigating the relationship between LTBI and helminths in a large, homogenous and well-controlled population of migrants from an endemic region relocating to a low burden setting. We hypothesized that the presence of helminths would have a negative impact on host functional immune responses to *M.tb*. However, we observed that asymptomatic hookworm infection was associated with reduced risk of *M.tb* infection. Further investigation of this relationship using an *in vitro* mycobacterial growth assay and whole blood transcriptomics showed that the presence of hookworm infection enhanced mycobacterial growth inhibition and was associated with an eosinophil-related transcriptomic signature. Our findings suggest that hookworm infection may influence the outcome to *M.tb* but this may depend upon the clinical status of the *M.tb* infection.

## Materials and Methods

### Study Population and Experimental Design

**Figure 1:**
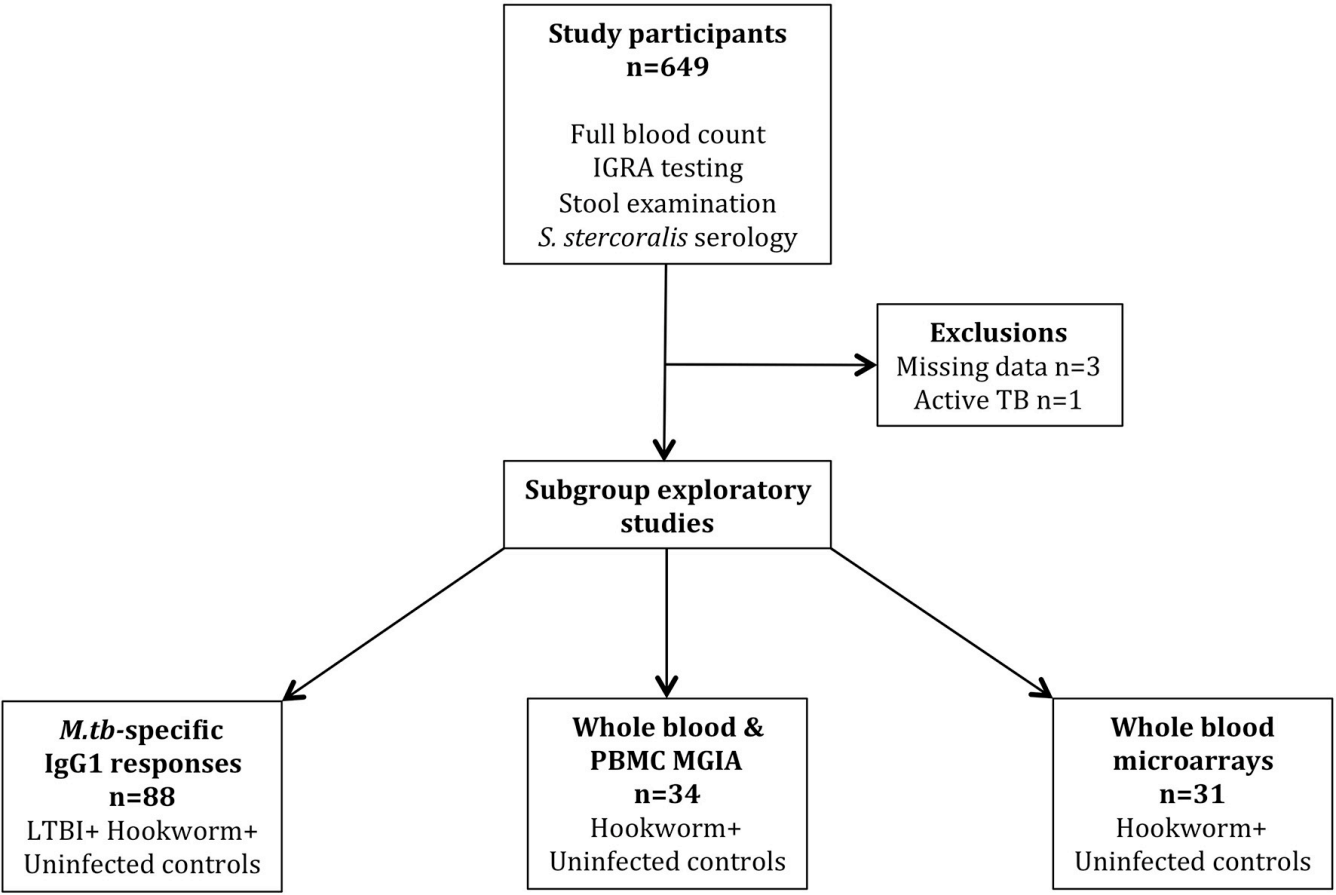
Study schedule.

This was a prospective, longitudinal cohort study of male adult (≥18 years) military recruits who had recently arrived in the UK from Nepal. The Nepalese vaccination policy for TB is to immunize once at birth with BCG and the latest estimates of TB incidence in Nepal are 154 (136–174) per 100,000 population (27). Participants were recruited between 2012 and 2015 and routine entry screening data were collected, including full blood count, stool analyses for gastrointestinal parasites (GIP), and serology for *Strongyloides stercoralis* infection. LTBI status was determined by IGRA testing using the T-SPOT.TB enzyme-linked immunospot (ELISPOT) assay (performed by Oxford Immunotec, Abingdon, United Kingdom). IGRA testing was repeated 3–6 months after the completion of treatment for GIP to assess its impact on IGRA conversion rates. In addition, an *M.tb*-specific ELISA, a functional mycobacterial growth inhibition assay (MGIA) and whole blood transcriptomic analysis were performed on peripheral blood samples from a subgroup of participants with hookworm infection and matched healthy controls. In accordance with current military and UK migrant screening policy, HIV status was not determined. The estimated prevalence of HIV infection among adults aged 15–49 years in Nepal is 0.2% (27). All participants provided written informed consent and ethical approval was granted by the Ministry of Defence Research Ethics Committee (MODREC 237/PPE/11). The study schedule is shown in Figure 1.

### Stool Analysis

Three separate fecal samples were received from each participant at weekly intervals. Fresh fecal samples were processed as follows: 1–2 g of feces was added to 10 ml of formalin water, mixed well by vortexing and left to stand for up to 3 min. Fecal concentration was performed using a modification to Ritchie's formol-ether method with Evergreen® fecal parasite concentrators (28). Following concentration, two 22 × 22 mm coverslips of sample were prepared and examined by light microscopy for the presence of ova, cysts, or larvae. Two experienced parasitology microscopists performed slide examinations independently (scanned using x100 magnification, with abnormalities scrutinized at x400). In addition, a fecal-charcoal culture was established for each sample using standard techniques (29). Following incubation for 6 days at 26°C, samples were examined for *S. stercoralis* or hookworm L3 larvae. Slides were also prepared from the formol-ether deposit and stained using the modified Ziehl Neilsen technique for the presence of cryptosporidium or other coccidian oocysts (30). Each slide contained two separate smears of fecal material and two different experienced microscopists examined one smear each. The presence or absence of parasitic infection was recorded. The presence of *Entamoeba* cysts was excluded from analyses because distinction between pathogenic (*E. histolytica*) and non-pathogenic (*E. dispar* and *E. moshkovskii*) species is not possible by microscopy due to morphological similarities.

#### *Strongyloides stercoralis* Serology

A sample of clotted blood was collected from each participant and following centrifugation, serum was aliquoted and stored at −20°C. Samples were thawed and total IgG antibodies to *S. stercoralis* were detected by ELISA using standard protocols (31). Samples were tested in duplicate at a screening dilution of 1:100 with a diagnostic threshold optical density (OD) of ≥0.20.

### Mycobacterial Culture and Titrations

*M.tb* H37Rv was obtained from BEI Resources (VA, USA). Frozen 1 ml aliquots of strain stocks were thawed and grown in 100 ml Middlebrook 7H9 media with 10% OADC to mid-log phase then divided into 1 ml aliquots and stored at −80°C until required. Mycobacterial stocks were titrated by serial dilution in supplemented 7H9 media and cultured in BACTEC MGIT tubes supplemented with PANTA antibiotics and OADC enrichment broth (Becton Dickinson, United Kingdom), as previously described (32). In addition, the number of colony forming units (cfu) for each dilution was determined by solid culture on 7H10 agar plates. A standard curve of time-to-positivity (TTP) against cfu was derived and linear regression analysis generated an equation to convert experimental TTP to cfu.

### MGIT Mycobacterial Growth Inhibition Assay

The whole blood direct MGIA was performed as previously described (33). Duplicate tubes containing 300 μl of whole blood were incubated with 300 μl of RPMI (containing 10% pooled human serum, 2 mM L-glutamine and 25 mM HEPES; Sigma, UK) inoculated with ~150 cfu *M.tb* on a 360° rotator at 37°C for 96 h (volume of the mycobacterial stock was calculated to give a TTP of 6.5 days, previously determined to give optimal differential responses). Cells were then lysed with sterile water, the mycobacteria resuspended in 7H9 media and transferred to a BACTEC MGIT tube supplemented with PANTA antibiotics and OADC enrichment broth (Becton Dickinson, UK). Tubes were placed in the BACTEC 960 machine and incubated at 37°C until the detection of positivity by fluorescence (TTP). In addition, on day 0, duplicate viability control tubes were set up by directly inoculating supplemented BACTEC MGIT tubes with the same volume of mycobacteria as the samples.

Peripheral blood mononuclear cells (PBMC) were separated from fresh heparinized whole blood by density gradient centrifugation, cryopreserved and thawed using standard techniques (34). The PBMC direct MGIA was as above but used 3 × 10^6^ cryopreserved PBMC in 300 μl of RPMI-MGIT media per culture, inoculated with ~10 cfu *M.tb* (estimated TTP of 8.5 days) and then incubated in 48-well tissue culture plates at 37°C for 96 h (34). Mean TTP for duplicates was converted to a cfu count (as described above) and net growth ratio was calculated as Log_10_(sample cfu/control cfu). A smaller net growth value indicates less bacillary replication and therefore represents greater mycobacterial control.

### Anti-*M.tb* IgG1 Enzyme-Linked Immunosorbent Assays

Microtiter plates were coated overnight at room temperature with a 1:1 mix of ESAT-6/CFP-10 (10 μg/ml each) prepared from *M.tb* H37Rv (BEI Resources). Native human IgG1 (2 μg/ml; AbD Serotec, UK) and PBS were included as positive and negative controls (respectively). After washing with PBS containing 0.05% Tween 20 (PBS/T) and blocking with casein, serum samples were tested in duplicate at 1:10 (diluted in casein). Casein was used for control wells. After incubation at room temperature, biotinylated secondary antibody (anti-IgG1; Life Technologies) at 1:1,000 dilution was added, followed by washing and the addition of Extravidin-ALP (1:5,000; Sigma Aldrich). pNPP substrate (Sigma Aldrich) was added and plates were read at 405 nm every 10 min using an ELx800 Microplate Reader with Gen5 software until the IgG1 isotype control reached an OD of 0.6 (previously determined from the gradient of a standard curve of native human IgG1 ranging from 0.1 to 100 μg/ml to avoid saturation). The mean OD of negative controls was subtracted from the mean OD of samples and readings with an OD > negative control plus three standard deviations (SD) were considered positive.

### Gene Expression Microarray Transcriptomics

Peripheral blood was collected in PAXgene tubes and stored at −80°C until required for whole blood transcriptomic studies as previously described (33). Samples were subsequently thawed at room temperature for 2 h, total intracellular RNA was extracted using the Blood RNA Kit (Qiagen) and the purity and quantity of RNA was assessed prior to storage at −20°C. Globin mRNA was depleted using the GLOBINclear Kit (Ambion), amplified and biotin-labeled using the TotalPrep RNA Amplification Kit (Illumina). RNA was purified and the quality assessed using an Agilent bioanalyser. Biotinylated cRNA was hybridized to Illumina HumanHT-12 (v4.0) expression beadchips according to the manufacturer's instructions. Beadchips were scanned with an Illumina iScan bead array reader confocal scanner and data extracted using GenomeStudio software.

### Microarray Analysis

Raw, probe level summary values as exported from Illumina GenomeStudio 2011 of Illumina HumanHT-12 (v4.0) microarrays were imported into R using beadarray (35). Probes were background corrected followed by quantile normalization using the neqc command (36). The analysis was restricted to probes with a detection *p* < 0.05 in at least 10% of the samples and probes matching to GENCODE version 26 with at most 2 mismatches. A linear model was fitted using limma to determine differential expression including parameters for sample collection batch effects, as well as unobserved batch effects, by the inclusion of the first three surrogate variables as provided by the R package sva (37, 38). Array quality weights were incorporated in order to account for between array quality differences (37, 39). To account for between-patient correlation, the duplicateCorrelation command from the limma package was used. All *p*-values were corrected for multiple hypothesis testing using the Benjamini-Hochberg procedure (40). The transcriptomic dataset has been deposited in NCBI's Gene Expression Omnibus (GEO) database and is accessible through accession number GSE122737.

### Statistical Analysis

Statistical analysis was performed using GraphPad Prism Software Version 6.0 (GraphPad, La Jolla, CA, United States) and SPSS Statistics Version 22.0 (IBM, United States). Specific statistical tests used are indicated in the results. For unsupervised clustering, one minus the Pearson correlation coefficient was used as dissimilarity metric to cluster samples, and complete linkage was used as the agglomeration rule.

## Results

### Hookworm Infection and Positive IGRA Responses Are Negatively Correlated

A total of 649 individuals participated in the study, of whom 645 were included in the final analyses (Figure 1). Exclusions were due to missing IGRA and/or stool results (*n* = 3) and a diagnosis of probable active TB during screening (*n* = 1, smear-negative pulmonary disease). All participants were male and from Nepal with a median age of 19.3 years (IQR 18.7–20.0 years). LTBI was diagnosed by IGRA positivity in 102/645 (15.8%) participants. The only difference in hematological parameters between LTBI-positive and -negative groups was significantly elevated eosinophil counts among LTBI-negative individuals (Table 1).

**Table 1:** Age, IGRA and hematological characteristics of the population by LTBI status.

**Characteristic**	**LTBI + Median [IQR]**	**LTBI − Median [IQR]**	*P*-value^*^
***n*** **=** **645**	***n*** **=** **102**	***n*** **=** **543**	
Age (years)	19.2 [18.6–19.9]	19.3 [18.7–20.0]	0.835
**ESAT-6 SFU/well**	**10 [4.0–25.3]**	**0 [0.0–1.0]**	**<0.0001**
**CFP-10 SFU/well**	**18 [9.8–36.3]**	**0 [0.0–1.0]**	**<0.0001**
***n*** **=** **414**	***n*** **=** **65**	***n*** **=** **349**	
Total leucocytes (x10^9^/L)	7.8 [6.6–9.6]	8 [6.7–9.4]	0.585
Neutrophils (x10^9^/L)	4.8 [3.6–6.1]	4.7 [3.7–6.1]	0.920
Lymphocytes (x10^9^/L)	1.9 [1.6–2.4]	2.1 [1.8–2.4]	0.092
Monocytes (x10^9^/L)	0.4 [0.4–0.5]	0.4 [0.4–0.6]	0.743
**Eosinophils (x10**^**9**^**/L)**	**0.2 [0.1–0.4]**	**0.2 [0.2–0.4]**	**0.002**
Basophils (x10^9^/L)	0.0 [0.0–0.1]	0.1 [0.0–0.1]	0.687
Hemoglobin (g/L)	150 [143–158]	152 [147–158]	0.291
Mean cell volume (fL)	90.0 [87.5–93.6]	90.1 [87.8–92.4]	0.323
Platelets (x10^9^/L)	236 [209–271]	250 [217–284]	0.118

* *Mann-whitney U-test; significant differences highlighted in bold*.

*LTBI, latent TB infection*.

*IQR, interquartile range*.

*SFU, spot forming unit*.

GIP infections were identified in 140/645 (21.7%) participants, among whom single, dual and triple infections numbered 115, 21, and 4 (respectively). *Giardia lamblia* was the most commonly identified parasite (54/169, 32.0% of infections), followed by hookworm (47/169, 27.8%) (Table 2). Quantification of GIP infection was not formally recorded, but generally only low burdens of infection were noted. There was no difference in GIP infection status stratified by LTBI status, except for a significant negative association between the presence of hookworm infection and a positive IGRA (OR = 0.221; 95%CI 0.05–0.93; *p* = 0.039) (Table 2). Further experiments were performed to assess whether this finding could be due to false-negative IGRA results in this group.

**Table 2:** Prevalence of GI parasite infection and association with IGRA positivity.

**Helminth/protozoan species *n* = 169**	***n***	**LTBI + *n* = 102 no. (%)**	**LTBI −*n* = 543 no. (%)**	***P*-value^**a**^**	**Univariate OR (95%CI)**	***P*-value**
*Ascaris lumbricoides^*b*^*	2	1 (1.0)	1 (0.2)	–	–	–
**Hookworm species**	**47**	**2 (2.0)**	**45 (8.3)**	**0.021**	**0.221 (0.05–0.93)**	**0.039**
*Hymenolepis nana^*b*^*	6	3 (3.0)	3 (0.6)	–	–	–
*Strongyloides stercoralis*	39	4 (4.0)	35 (6.4)	0.495	0.592 (0.21–1.71)	0.332
*Trichuris trichiura*	21	2 (2.0)	19 (3.5)	0.555	0.552 (0.13–2.41)	0.428
*Giardia lamblia*	54	7 (6.9)	47 (8.7)	0.697	0.778 (0.34–1.77)	0.550
Any helminth/protozoan	169	19 (18.6)	150 (27.6)	0.066	0.6 (0.35–1.02)	0.06

a *Fisher's two-tailed LTBI+ vs. LTBI-; significant differences highlighted in bold*.

b *Statistical analyses were not performed where total parasite infection number was <10*.

*LTBI, latent TB infection; OR,odd's ratio; CI, confidence interval*.

### Treatment of Hookworm Infection Does Not Promote IGRA Conversion

**Figure 2:**
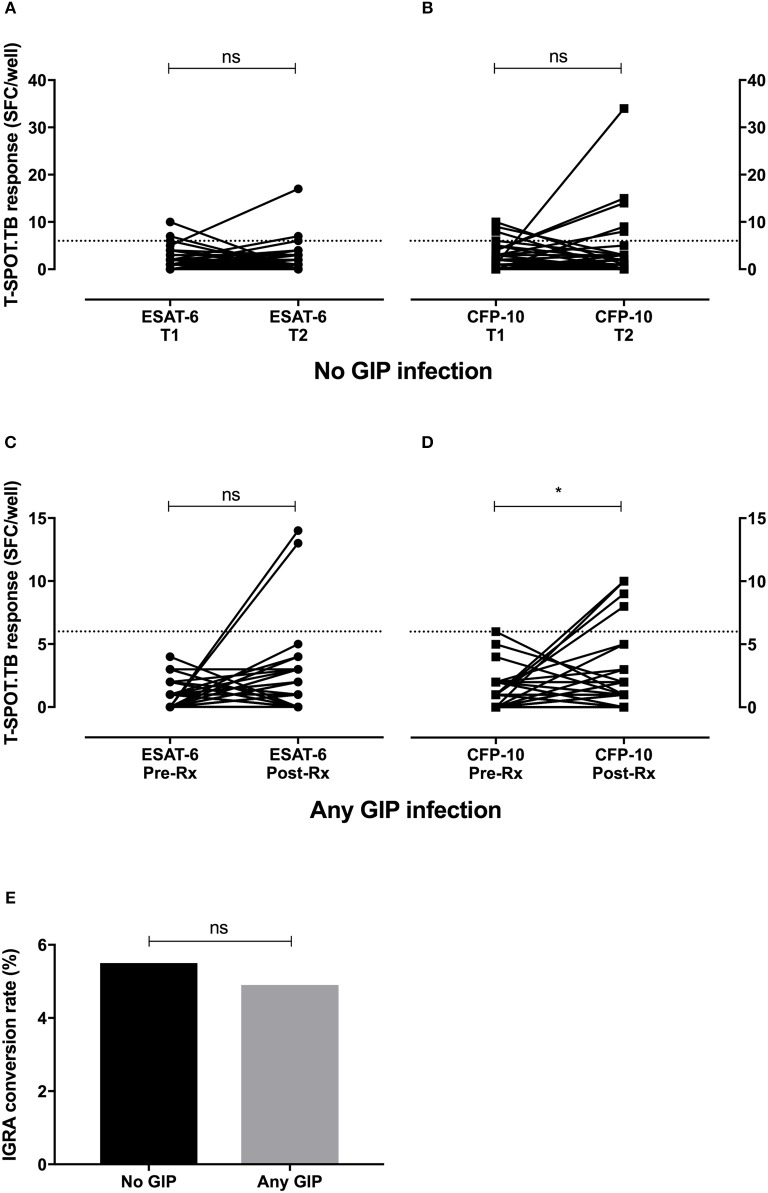
Treatment of hookworm infection does not promote IGRA conversion to positivity. Longitudinal IGRA responses were assessed among individuals with a negative IGRA at baseline (*n* = 212). IGRA testing was repeated after either no intervention (no GIP infection, *n* = 110, **A,B**) or after GIP treatment (any GIP infection, *n* = 102, **C,D**). While there were significant increases in some T cell responses to *M.tb*-specific antigens, there was no significant difference in the conversion rate to IGRA positivity between the groups **(E)**. GIP, gastrointestinal parasite; SFC, spot forming cells; T, time point; Rx, treatment for GIP; gray dashed line, threshold for a positive IGRA; ns, non-significant; *p < 0.05.

If hookworms have a modulatory anti-Th1 effect then it could be expected that IGRA-positivity would increase after treatment of infection. To examine this we assessed IGRA conversion to positivity in a subgroup of individuals with a negative IGRA at baseline (*n* = 212). Among this group, 110 volunteers did not have any evidence of GIP infection, while ≥1 GIP was identified in 102 individuals (including 38 with hookworm). IGRA testing performed 3–6 months after either no intervention (for individuals without GIP infection, termed controls) or completion of GIP treatment showed six conversions to positivity in those without a GIP (5.5%), and five conversions in the group with any GIP (4.9%) (Figure 2). There was no significant difference in the rates of IGRA conversion between the groups (*p* = 1.0, two-tailed Fisher's exact test). Therefore, in our cohort the treatment of GIPs did not significantly alter IGRA conversions or change the number of LTBI diagnoses above the background rate.

### *M.tb*-Specific Antibody Responses Are Significantly Higher in Individuals With LTBI Than in Those With Hookworm Infection

**Figure 3:**
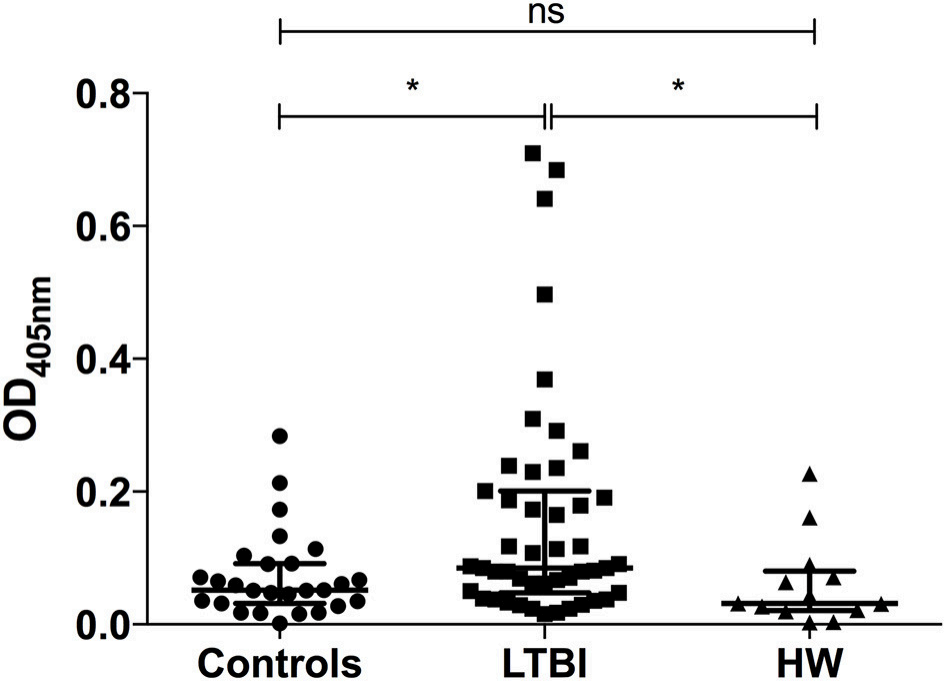
IgG1 responses to *M.tb*-specific antigens are significantly different between individuals with LTBI and those with hookworm infection. Antigen-specific IgG1 responses to ESAT-6/CFP-10 in pre-treatment serum samples from uninfected healthy controls (*n* = 28), and individuals with either LTBI (*n* = 47) or hookworm infection (*n* = 13) were determined by ELISA. Optical densities (ODs) are reported following subtraction of the background. Points represent the mean of duplicates and bars are the median values with the interquartile range. After normality testing a Kruskal-Wallis test with Dunn's correction for multiple comparisons was performed. ns, non-significant; *p < 0.05. LTBI, latent TB infection; HW, hookworm.

We have previously seen that IgG1 responses to certain *M.tb*-specific antigens discriminate between individuals with LTBI and uninfected controls (41). To identify whether humoral responses to *M.tb* also differed between the helminth-infected and non-infected groups, we measured IgG1 levels against ESAT-6 and CFP-10, two antigens present in *M.tb* but not BCG, in serum from uninfected healthy controls (*n* = 28), and individuals with either LTBI (*n* = 47) or hookworm infection (*n* = 13). Antibody responses were significantly higher in the LTBI group (median OD_405_ = 0.085) compared to the control and hookworm groups (median OD_405_ = 0.052 and 0.032, respectively; Figure 3), consistent with the results of IGRA testing.

### Individuals With Hookworm Infection Have Improved *in vitro* Mycobacterial Control Which Is Reversed After Helminth Treatment

**Figure 4:**
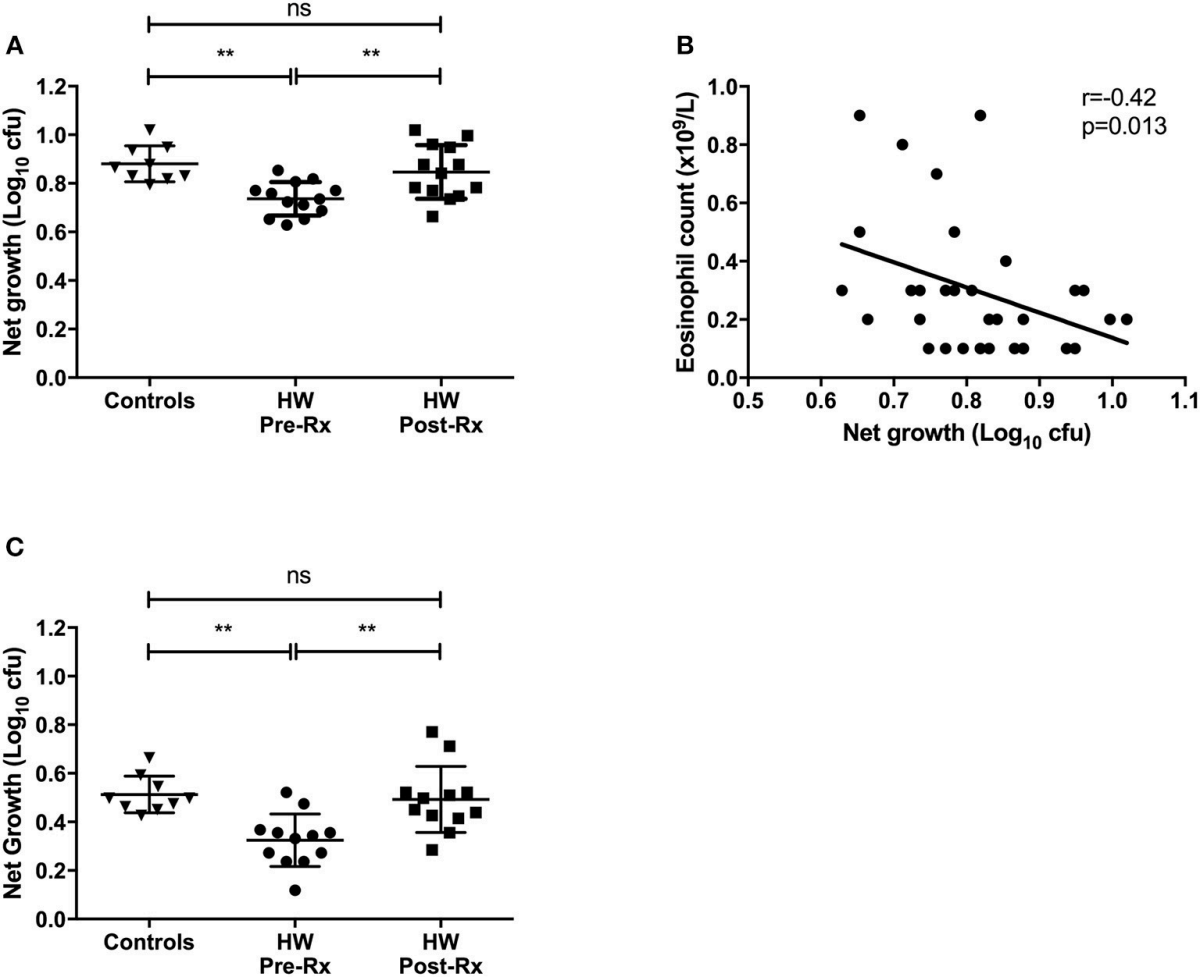
*In vitro* differential mycobacterial control is improved among individuals with hookworm infection in whole blood and PBMC and is lost after helminth treatment. Whole blood and PBMC MGIT using *M.tb* H37Rv was performed to assess mycobacterial control. Whole blood samples were available from uninfected controls (*n* = 9) and matched pre- and post-treatment samples from hookworm infected individuals (*n* = 13) **(A)**. The relationship between mycobacterial net growth and hematology parameters was investigated. After testing for normality, Spearman's correlations were calculated between hematology data and all whole blood *M.tb* H37Rv MGIT results (*n* = 34) and showed a significant negative correlation growth and eosinophil count **(B)**. MGIA was also performed using cryopreserved PBMC with matched pre- and post-treatment PBMC samples from individuals with hookworm infection (*n* = 12) and controls (*n* = 9) **(C)**. For MGIT data, points represent the mean of duplicates; bars represent mean values with SD. A one-way multiple comparison ANOVA with Tukey's post-test correction was performed between the groups. HW, hookworm; Rx, treatment; ns, non-significant; **p < 0.005.

The functional ability of an individual to control growth of virulent mycobacteria was assessed in the direct MGIA using samples before and after successful treatment for hookworm infection. Whole blood samples were available from 9 uninfected controls and matched pre- and post-treatment samples from 13 individuals with hookworm infection. Growth of *M.tb* was significantly lower in pre-treatment hookworm whole blood compared to controls (mean net growth: 0.74 vs. 0.88 log_10_ cfu, *p* = 0.0019; Figure 4A). Mycobacterial net growth increased significantly after treatment for hookworm infection (mean net growth = 0.85 log_10_ cfu, *p* = 0.0086), to levels comparable to the controls. Whole blood contains several cellular constituents that may have anti-mycobacterial functions, therefore we assessed which elements correlated with the mycobacterial growth rate using all samples and each time point (*n* = 34). The only significant correlation seen was a negative relationship between net growth and eosinophil counts (rho = −0.42, *p* = 0.013; Figure 4B). Finally, we repeated the MGIA using cryopreserved PBMC (lacking eosinophils). Matched pre- and post-treatment PBMC samples were available from 12 individuals with hookworm infection and baseline samples from 9 controls. A pattern similar to the whole blood experiments was seen, with reduced mycobacterial growth in individuals before treatment for hookworm, which increased following treatment (mean net growth: 0.32 vs. 0.48 log_10_ cfu, *p* = 0.0027; Figure 4C). Therefore, the *in vitro* ability to control growth of *M.tb* was improved among individuals with hookworm infection and this control was associated with higher eosinophil counts in whole blood.

### Eosinophil-Associated Transcriptomic Profiles in Whole Blood Correlate With Improved Mycobacterial Control

**Figure 5:**
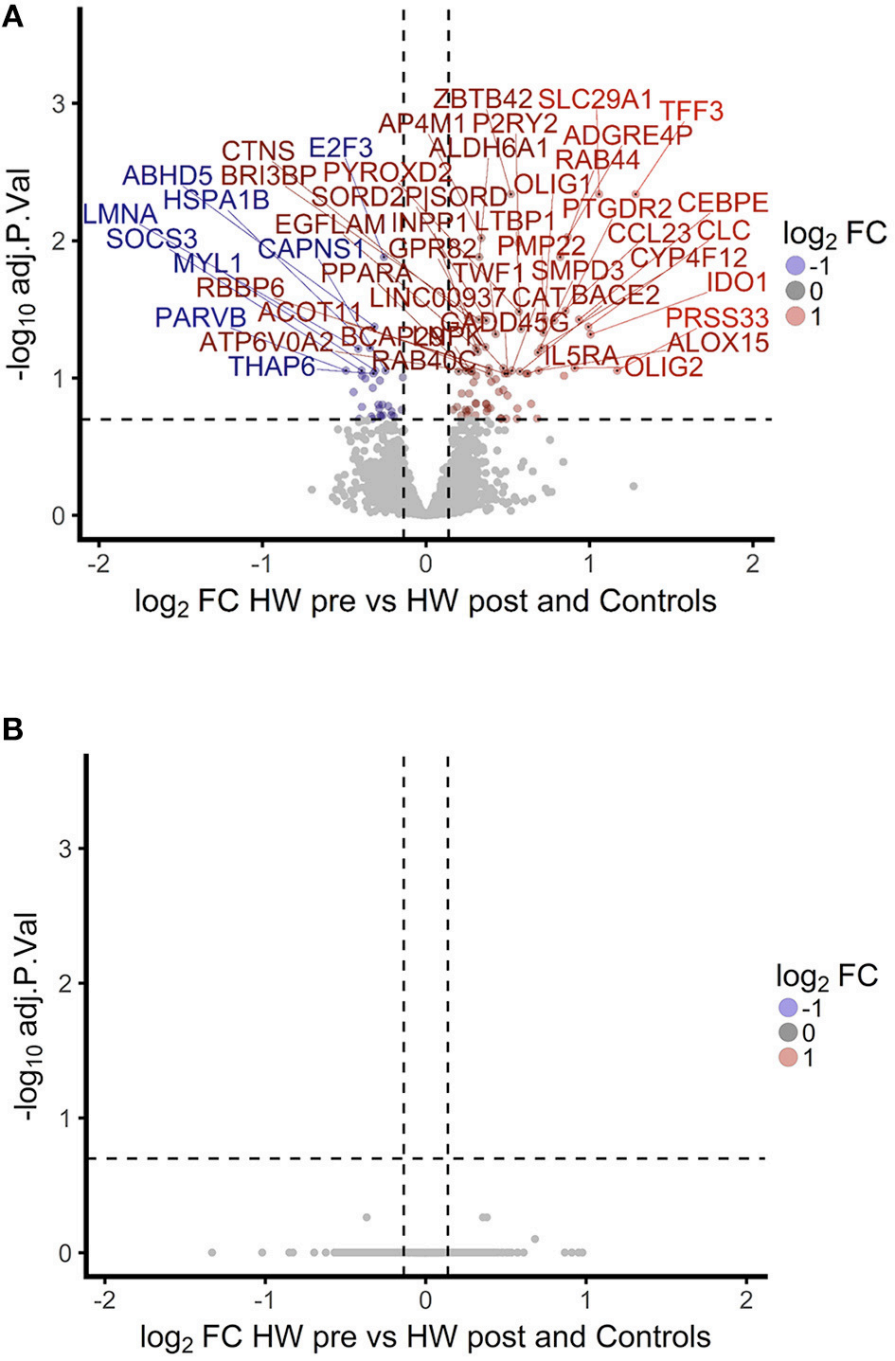
A hookworm-specific transcriptional signature comprising 112 significant probes was detected in infected compared to treated and uninfected individuals and adjustment for eosinophil count leads to loss of the hookworm-specific signature. **(A)**. Volcano plot showing magnitude and significance of differential expression between hookworm infected volunteers before and after treatment. Infected volunteers post-treatment were analyzed in combination with a hookworm naïve control group to increase power. Gene names of the top 50 probes with an FDR below 20% are shown above the dashed line. FC, fold-change; HW, hookworm; pre and post refer to treatment. **(B)**. Volcano plot showing the fold changes between hookworm infected volunteers before and after treatment. The linear model contained eosinophil counts as an additive term to adjust for differences in eosinophils. No probe reached significance at 20% FDR. FDR, false discovery rate.

**Figure 6:**
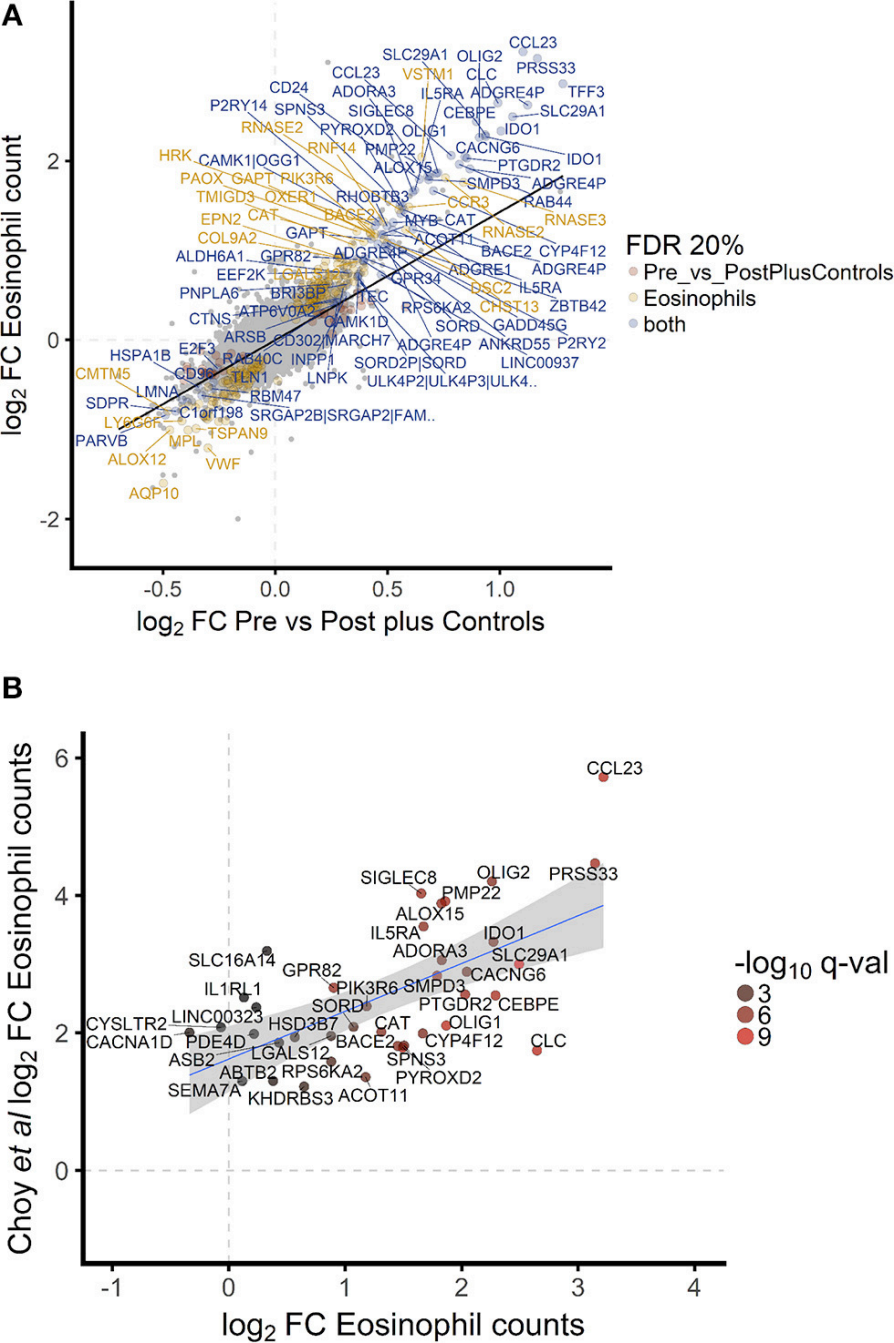
Hookworm-specific transcriptional signature is highly correlated to transcriptomic correlates of eosinophil count and is consistent with previously published signatures. **(A)**. Scatterplot of log2 fold changes of hookworm infected vs. post treatment and control samples. A regression line was added to highlight the correlation of the two contrasts and gene names of the top 100 differentially expressed probes were added. Point color indicates the 302 and 112 significantly differentially expressed probes below 20% FDR in hookworm infected and eosinophil counts, respectively. Seventy four probes are significantly differentially expressed in both contrasts. **(B)**. Of the top 50 genes described to be significantly associated with eosinophils based on whole blood gene expression in a recent publication (42), 40 were detected in our study and the gene names of all probes representing these genes are indicated.

**Figure 7:**
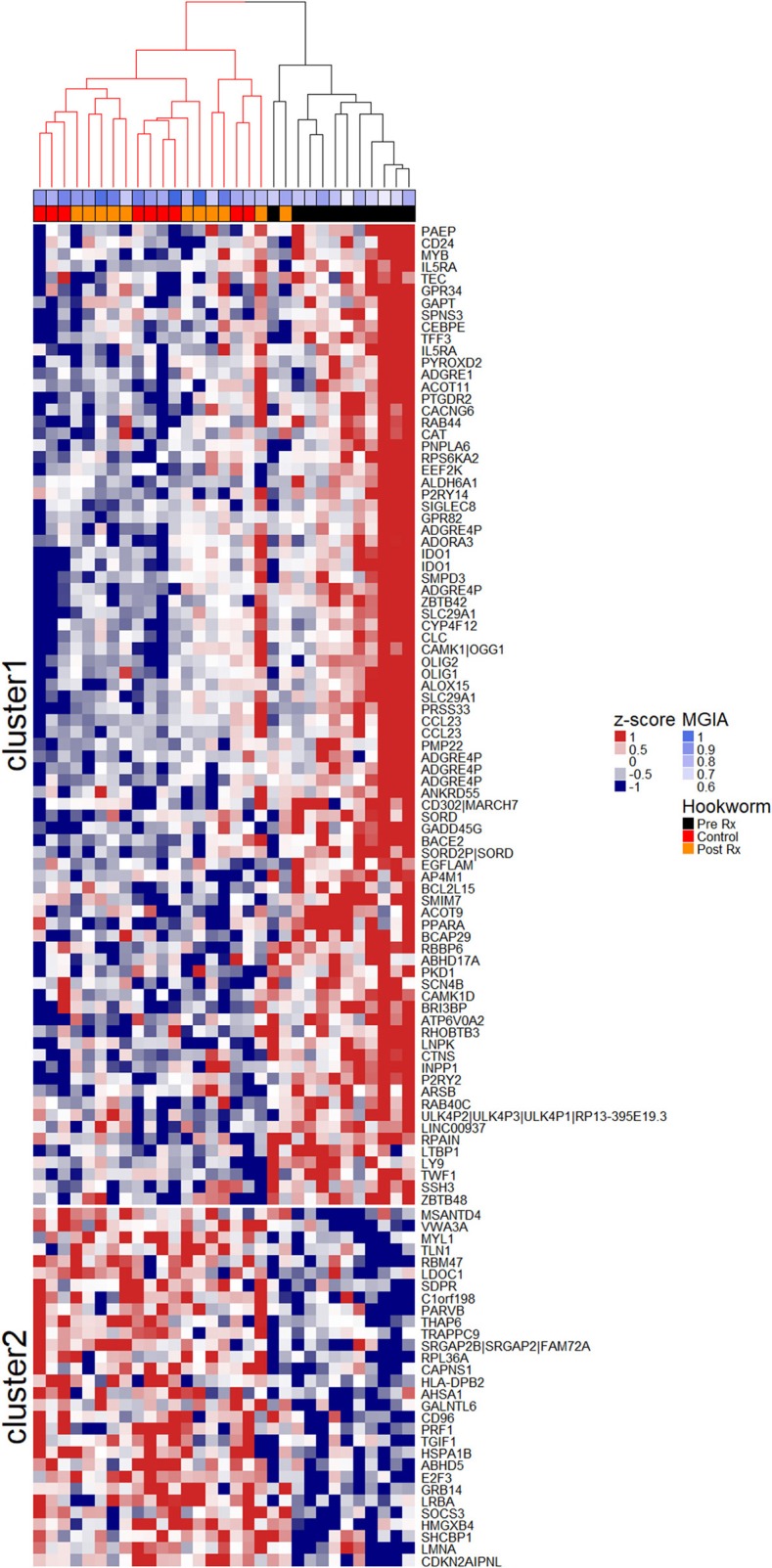
Unsupervised clustering of the hookworm-specific transcriptional signature reveals correlation to mycobacterial growth inhibition. Normalized expression values of 112 probes mapping to 104 genes were mean centered and scaled and columns were clustered using hierarchical clustering as described in the Materials and Methods. The two largest clusters of patients were colored in red and black and the two most distinct clusters of probes were separated into cluster 1 and cluster 2 by k-means clustering. MGIA control was significantly improved (*p* < 0.003) in the black cluster compared to the red cluster. A Welch Two Sample *t*-test was performed between groups. Rx, treatment.

To investigate the association between increased eosinophil counts and improved *M.tb* control in the blood of hookworm infected individuals, we performed an unbiased assessment of the impact of hookworm infection on whole blood by transcriptomics. Transcriptomics was performed on unstimulated whole blood samples from uninfected controls (*n* = 9) and individuals with hookworm infection (matched pre- and post-treatment, *n* = 11). We identified 112 significant probes, mapping to 104 genes, specific to individuals with hookworm infection before treatment (Figure 5A). Adjustment for eosinophil counts resulted in the complete loss of this hookworm-specific signature, suggesting that eosinophils are the primary contributor to the transcriptomic profile (Figure 5B). This was supported by the strong correlation of the transcriptomic changes to eosinophil counts and the changes following treatment of hookworm infection (Figure 6A). Further analysis of the signature showed it to be consistent with previous published studies of eosinophil-associated gene expression (Figure 6B). Finally, we performed unsupervised clustering analysis of the hookworm-specific transcriptional signature (Figure 7). Clustering of the expression data by columns separated the samples into two broad clusters. The black cluster which contained all 11 pre-treatment hookworm samples, showed a distinct cluster as compared to the red cluster which contained 19 of the 20 control and hookworm post-treatment samples. Mycobacterial growth control as determined by whole blood *in vitro* MGIA was significantly improved (*p* < 0.003) in the black pre-treatment cluster as compared to the red cluster.

## Discussion

Despite the huge global burden of LTBI and intestinal parasites, and their geographical overlap, there are few robust studies examining the relationship between them. We have performed a prospective study in a large well-defined cohort of adults and show, for the first time, that hookworm infection is negatively associated with LTBI and also with improved control of mycobacterial growth *in vitro*.

An important element of our findings, which challenge current dogma, is consideration of how intestinal helminth infections may affect diagnostic tests for LTBI. The T cell-based IGRAs rely on *M.tb*-specific antigens to stimulate effector T cells, eliciting the secretion of IFN-γ as an indicator of infection. The helminth-induced polarization of the immune axis postulated by several investigators may induce a Th2 response and therefore result in false-negative IGRA results. To date, studies have only shown an association between indeterminate IGRA results and helminths in pediatric populations using ELISA-based Quantiferon (QFT) IGRA tests. Indeterminate QFT results mainly occur due to failure of the positive mitogen control and are significantly associated with younger age, iron deficiency, and malnutrition (23–26). Malnourished individuals have reduced numbers of T cells and their function is impaired, resulting in reduced IFN-γ production in both human and animal studies (43–45). Therefore, indeterminate IGRAs could represent the direct effects of malnutrition, which may be independent of helminth infection. These confounders were not a feature in our population of healthy, asymptomatic young adults.

We sought to investigate this further and present evidence suggesting that helminth-induced false negative IGRA results did not occur in our cohort. Firstly, there was no significant difference in IGRA conversions to positivity among individuals following successful treatment for hookworm compared with the background rate in uninfected controls. If the IGRA results were falsely negative due to a hookworm-induced Th2 shift, it is plausible that restoration of unencumbered Th1 responses after treatment would result in excess IGRA conversions. We suggest that the absence of such phenomena during longitudinal assessment is further evidence that IGRA testing was not confounded by hookworm infection. Moreover, in a mouse model of helminth and Salmonella infection, helminths did not modulate Th1 responses (46). Secondly, humoral responses may have a role in serodiagnostics for TB and therefore we assessed antibody responses (47–49). We have previously shown a strong negative association between IgG1 responses to the RD1-restricted protein antigens ESAT-6/CFP-10 and *in vitro* mycobacterial growth among individuals with LTBI and active TB disease, reflecting bacillary burden and therefore disease status (41). In this study, we found comparably low antibody responses to ESAT-6/CFP-10 among uninfected controls and individuals with hookworm that, in the context of a negative IGRA, we contend reflects the absence of LTBI.

Finally, we have shown that prior to treatment for hookworm, infected individuals demonstrate improved growth control of virulent *M.tb* in both a whole blood and PBMC MGIA, and this ability is lost after successful treatment. Previous studies of the relationship between TB and helminth infections have measured individual immune parameters, such as cytokine secretion by antigen-specific T cells. However, given the complexity of these pathogens such an approach may be over simplistic. In contrast, *in vitro* MGIAs are functional assays that provide a summative assessment of a range of immune mechanisms and their complex interactions within a biological sample (50). In the absence of defined mechanisms, this assay provides an unbiased assessment of mycobacterial control as a measure of anti-mycobacterial immunity. The novel findings we have described using this assay are consistent with our immuno-epidemiological observations. It was not possible to include an LTBI-positive, hookworm-negative control group in either the whole blood or PBMC-based MGIA in this study. However, we have recently described such a population using an *M.tb* whole blood MGIA and as we use standard controls and report the functional output as a growth ratio, these data are comparable between studies (41). In that study the growth control of H37Rv among individuals with evidence of LTBI (IGRA-positive) was enhanced in relation to uninfected healthy controls, which we suggested was due to increased immune activation in response to bacillary burden. The *M.tb* growth rate in pre-treatment hookworm infected individuals in this study was less than that observed previously among those who were LTBI-positive (hookworm-negative). While speculative, we again propose that this finding may arise from differential immune activation and possibly suggests a contribution from innate cells not identified in previous cohorts (e.g. eosinophils).

The validity of our findings is supported by the reversal of improved control following treatment for hookworm. While assessment of the mechanism was beyond the scope of this study, we hypothesize that eosinophils may contribute to this control for several reasons. Firstly, the only difference in hematological parameters between groups based on IGRA status was increased eosinophil counts among those negative for LTBI. Secondly, the transcriptomic profile of whole blood from hookworm infected individuals was dominated by an eosinophil-associated signature that did not persist after normalization of eosinophil counts (either after hookworm treatment or by correction during microarray analysis). Some of the significantly expressed genes we identified that have been associated with eosinophils included PRSS33, CCL23, IL5RA, and SIGLEC8. PRSS33 (protease serine 33) is a single hydrophobic-domain transmembrane protein constitutively expressed in human eosinophils. Cell surface expression of PRSS33 is associated with fibroblast extracellular matrix protein synthesis in activated eosinophils (51). CCL23, a novel C-C chemokine, is a CCR1 agonist with chemotactic activity on monocytes, dendritic cells, and resting T lymphocytes, together with roles in the potentiation of VEGF-induced proliferation and migration of human endothelial cells. Eosinophils have been shown to produce and release CCL23 following stimulation with GM-CSF or IL5 (52). The alpha subunit receptor for IL5 (IL5RA), which is IL5-specific, was another highly expressed gene we identified that correlated significantly with eosinophils. IL5 is the principal cytokine involved in the maturation, proliferation, and activation of eosinophils (53). Finally, SIGLEC8 (sialic acid binding Ig-like lectin 8) is a single pass transmembrane inhibitory receptor predominantly expressed on the surface of human eosinophils which, following the binding of specific glycans, induces eosinophil apoptosis (54). Clinically, SIGLEC8 may play a role in preventing chronic lung inflammation in asthma by inducing cell death of activated eosinophils, supported by the observation that polymorphisms have been associated with asthma susceptibility in humans (55, 56). Collectively, the dominant differential expression of these and other genes, and their correlation with eosinophil counts, provide evidence for an eosinophil-specific transcriptomic profile which is also consistent with the findings of other investigators (42, 57). Thirdly, the number of eosinophils in whole blood was the only cellular constituent to correlate with mycobacterial growth *in vitro*. Interestingly, a recent study has also shown a negative correlation between mycobacterial growth rate and eosinophil count in blood from African buffalo (*Syncerus caffer*) (58). Eosinophils have an important role in innate immune responses, particularly against helminths, and while typically present in low numbers, may make a significant contribution to the proportion of blood leucocytes when elevated (indeed, in our hookworm infected cohort eosinophils accounted for up to 14% of total leucocytes and 23% of polymorphonuclear cells) (59). We suggest that hookworm-induced eosinophilia may have a role in defense against *M.tb*, supported by studies from other investigators showing that these cells are activated by mycobacteria, accumulate at sites of mycobacterial infection and have anti-mycobacterial activity through the secretion of cytotoxic proteins (60–63).

However, this study was not designed to formally assess the possible direct and/or indirect mechanisms of eosinophil activity against *M.tb*. For example, apart from the direct anti-mycobacterial action of eosinophils to control *M.tb* growth, the indirect action of Th2-mediated control of lung tissue damage may modulate susceptibility to *M.tb* infection. We acknowledge that our findings are of association rather than causation and accept that quantitative differences in eosinophil counts may not result in qualitative differences in eosinophil function; further studies are required. In addition, we do not suggest that eosinophils are the sole mediators of mycobacterial control, as evidenced by the PBMC-based MGIA in which reduced growth of *M.tb* in hookworm infected individuals was seen in the absence of polymorphonuclear cells. Differential adaptive immune responses are likely to be important and as the number of PBMC has been standardized in this assay, it suggests that anti-mycobacterial control is due to qualitative rather than quantitative factors, consistent with other studies (64).

Furthermore, consideration of helminths with a pulmonary migratory phase, such as hookworm, adds to the biological plausibility of our findings. Studies show that even a transient exposure to hookworm not only recruits innate cells to the lungs (both eosinophils and alternatively activated macrophages), and induces changes in T and B cells, but can also produce long-lived alterations in the pulmonary immune environment that may have a role in enhancing subsequent responses to respiratory pathogens, including *M.tb* (18, 65–67).

The strengths of our study are predominantly due to the size and nature of the population, together with the study setting. Participants were matched for age, sex, and ethnicity and both helminth and *M.tb* exposure was controlled for as study participants were confined to a military training facility in a non-endemic setting. Assessment of GIP infection and LTBI status was comprehensive. Limitations include a relatively small number of hookworm infected individuals for subgroup analysis and that our population was extremely fit and healthy with low burdens of GIP infection. These individuals are unlikely to be representative of typical migrants relocating to low endemic regions.

In summary, we have shown that in a healthy young adult group of migrants arriving in the UK, asymptomatic hookworm infection is negatively associated with the presence of *M.tb* infection and with improved *in vitro* control of mycobacteria, which may be partly attributable to number and/or function of eosinophils. Further studies of the possible role of eosinophils in this context are warranted to investigate this further. Finally, these findings may have implications for mass drug administration (MDA) programmes intended to reduce or eliminate soil-transmitted helminths, including hookworm. The benefits of such programmes in reducing malnutrition and anemia and possible improvement in cognitive function of children continue to be debated, particularly for hookworm infections, which affect both children and adults in endemic settings (6, 68, 69). The effect of MDA programmes on the prevalence and severity of TB and the success of TB control programmes has not, to our knowledge, been systematically investigated.

## Author Contributions

MKO and TEF: Conception and design; MKO, RT, MM, JWB, and SGS: Experimental work; MKO and JM: Analysis and interpretation. All authors drafting the manuscript for important intellectual content.

### Conflict of Interest Statement

The authors declare that the research was conducted in the absence of any commercial or financial relationships that could be construed as a potential conflict of interest.
